# Evolution of Epstein–Barr Virus Infection Seroprevalence in a French University Hospital over 11 Years, Including the COVID-19 Pandemic, 2013–2023

**DOI:** 10.3390/microorganisms13040733

**Published:** 2025-03-25

**Authors:** Aurélien Aubry, Catherine Francois, Baptiste Demey, Marie Louchet-Ducoroy, Christine Pannier, Christine Segard, Etienne Brochot, Sandrine Castelain

**Affiliations:** 1 Laboratoire de Virologie, Centre Hospitalier Universitaire Amiens Picardie, 80054 Amiens, France; aubry.aurelien@chu-amiens.fr (A.A.); francois.catherine@chu-amiens.fr (C.F.); demey.baptiste@chu-amiens.fr (B.D.); louchet-ducoroy.marie@chu-amiens.fr (M.L.-D.); pannier.christine@chu-amiens.fr (C.P.); segard.christine@chu-amiens.fr (C.S.); brochot.etienne@chu-amiens.fr (E.B.); 2 UR 4294, Agents Infectieux, Résistance et Chimiothérapie (AGIR), Université de Picardie Jules Verne, 80000 Amiens, France

**Keywords:** EBV, seroprevalence, COVID, primary infection, France

## Abstract

*Epstein–Barr virus* (EBV) is one of the most prevalent human viruses worldwide. The COVID-19 pandemic, with its social distancing measures, has disrupted the circulation of many viruses. Delayed EBV primary infection is known to increase the risk of secondary conditions, including infectious mononucleosis, multiple sclerosis, and Hodgkin’s lymphoma. In this context, we aimed to investigate whether EBV seroprevalence has been affected over time, particularly in relation to the COVID-19 period, by analyzing all patients admitted to Amiens University Hospital from January 2013 to December 2023 who underwent EBV serology. During this period, 19,771 EBV serologies were performed and analyzed. The total seropositive rate of EBV infections approached 90%, considering all non-negative serological profiles, with the rate stabilizing after 2017. The number of EBV serologies increased significantly until 2016, as well as the age of the screened patients. Less than 3% of patients remain seronegative after 25 years, indicating a seroprevalence of around 97%. The overall primary infection rate was 2.6%. There was no significant difference in the number of primary infections in 2020–2021, the years associated with confinements and curfews in France in the context of the COVID-19 pandemic, compared with the other years. The overall EBV seroprevalence and age of primary infection remained stable during the study period, suggesting a moderate impact of the COVID-19 pandemic on seroprevalence in this cohort.

## 1. Introduction

*Epstein–Barr virus* (EBV), also known as *human herpesvirus 4* (HHV-4), is among the most common human viruses. It is responsible for infections that generally occur in childhood, with transmission via the oral route [1]. In immunocompetent hosts, primary infection (PI) with EBV is usually associated with few or no symptoms in childhood or may lead to the development of infectious mononucleosis (IM) in adolescents or young adults. However, in certain instances, it can cause particular cancers, such as Hodgkin’s lymphoma, Burkitt’s lymphoma, gastric cancer, nasopharyngeal carcinoma, and diffuse large B-cell lymphoma [2,3].

EBV infection is prevalent worldwide, and approximately 90% of all adults show serological antibody positivity [4]. The seroprevalence among children varies depending on socioeconomic status, sanitary conditions, and educational level [5]. In recent decades, several studies have reported epidemiological changes in EBV infections. A study conducted in the USA showed that the seroprevalence of 6-to-19-year-olds declined from 72% in 2003–2004 to 65% in 2009–2010 [6]. In Japan, the prevalence of EBV decreased to 59% for 1995–1999 from >80% before early 1990 [7]. Another study in a French hospital showed that EBV infection decreased over 15 years and that seronegative rates increased from nearly 50% during 2001–2005 to >60% during 2011–2015 for patients aged <10 years [8]. In the Naples region of Italy, the EBV seroprevalence was 65% from 2007 to 2017 [9].

Similar studies were performed in China and showed a prevalence of EBV infection in North and South China of 80.78% and 79.38% for children aged 0–10 years, respectively [10], and more recently, a total seropositive rate of EBV infection of 61.02% among 44,943 children aged 0–18 years between January 2019 and December 2021 [11]. In this context, such an observed age shift in these countries could lead to a higher incidence of symptomatic EBV PI, in particular, intense IM. Different antibody patterns are used to distinguish acute from past EBV infection [1]. Understanding EBV seroprevalence trends is crucial, as the age at primary infection is directly associated with the risk of EBV-related diseases. In many developing countries, primary EBV infections occur at an earlier age due to different hygienic and social conditions [12,13,14], whereas delayed primary infection is associated with an increased risk of infectious mononucleosis and, consequently, a higher risk of Hodgkin’s lymphoma [15] or multiple sclerosis [16,17].

The COVID-19 pandemic has had a significant impact on many aspects of public health, including the transmission dynamics of other viral infections. Social distancing measures and lockdowns during the pandemic drastically reduced social interactions, including those between children. Personal protective measures included wearing masks, practicing hand hygiene, and maintaining social distancing.

Specific studies on the impact of the COVID-19 pandemic on EBV seroprevalence are still limited, but general trends in viral infections showed reduced transmission rates for many childhood viruses due to control measures of COVID-19. The suggestion of a decline in primary EBV infections [11] during this period raises concerns about a possible change in the timing of seroconversion. Given the potential long-term public health consequences, including risks for Hodgkin’s lymphoma and multiple sclerosis, further investigation of the epidemiology of EBV seems relevant. In addition, because seroprevalence can guide research into vaccines and therapies to prevent or treat EBV infections, it will be important to determine the evolution of seroprevalence during the last 10 years and whether the COVID-19 pandemic has had a significant impact on EBV transmission [18,19]. The aim of this study was to determine the seroprevalence of EBV infection over an 11-year period, including during the COVID-19 pandemic, and to assess potential changes in seroprevalence and in the age of primary infections, taking into account the implications of delayed seroconversion.

## 2. Materials and Methods

### 2.1. Data Collection

All outpatients and inpatients admitted to the Amiens University Hospital between January 2013 and December 2023 who underwent EBV serology were included. EBV serology was most commonly requested in the following clinical contexts: (i) diagnosis of infectious mononucleosis, (ii) evaluation of immunocompromised patients, or (iii) suspicion of EBV-associated pathology. EBV detection has been incorporated into several protocols for the management of immunocompromised, hematological, and oncological patients [20,21]. Inclusion criteria included all patients, regardless of pathology or prescribing clinical department. The only exclusion criteria were based on deduplication; if a patient had a previous EBV serology, only the first test result was retained unless seroconversion occurred, in which case both the first seronegative and the first seropositive results were retained. Demographic data, such as age and sex, were obtained from the medical records, as well as the date of serological testing.

### 2.2. Detection of EBV Antibodies

In this study, we focused on EBV seroepidemiology by examining the distribution and prevalence of EBV antibodies, such as VCA (Viral Capsid Antigen) IgM, VCA IgG, and EBNA (Epstein–Barr Nuclear Antigen) IgG, in particular, to assess the rates of primary infection. For all sera collected during this period, the detection of VCA IgG, VCA IgM, and EBNA IgG was performed using a two-step quantitative chemiluminescent immunoassay (Liaison^®^ system, Diasorin, Saluggia, Italy). Specific antibodies are captured by magnetic microparticles coated with synthetic peptides from VCA (p18) or EBNA-1 proteins. Detection is performed with anti-Human IgM or anti-Human IgG linked to an isoluminol derivative. For quantification of VCA IgM, a solution containing anti-Human IgG is added to increase the specificity of the VCA IgM detection. Antibody titers are then calculated using calibration curves, and the results are expressed in units per milliliter (U/mL). The applied cut-offs for the Liaison^®^ assays were 40 U/mL for VCA IgM, 20 U/mL for VCA IgG, and 20 U/mL for EBNA IgG [22]. In addition, doubtful results were interpreted using an algorithm proposed by DiaSorin (Saluggia, Italy): VCA IgM levels between 20 and 40 U/mL were considered positive if EBNA IgG levels were below 5 U/mL, and EBNA IgG levels between 5 and 20 U/mL were interpreted as positive if VCA IgM levels were below 20 U/mL [23].

**Figure 1:**
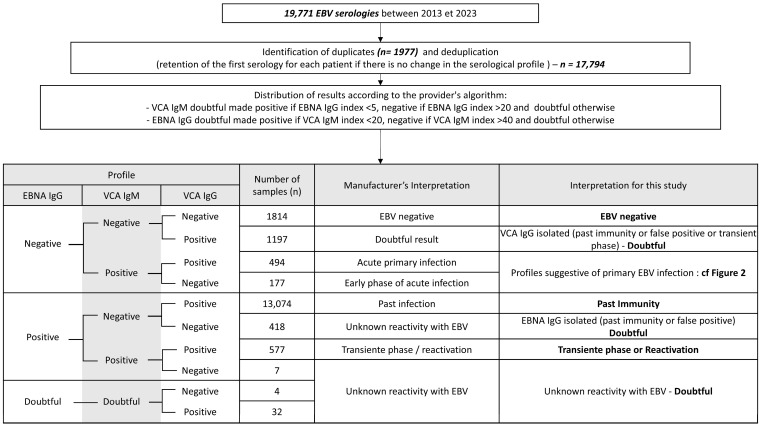
Flowchart of participant inclusion and EBV serology interpretation.

**Figure 2:**
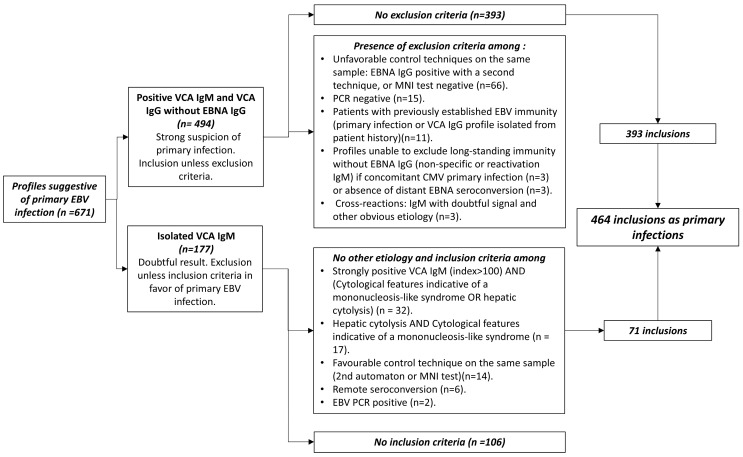
Flowchart of inclusion and exclusion criteria for defining a primary infection.

The EBV infection status of patients was classified according to the antibody pattern following the manufacturer’s recommendations (Figure 1). Primary infection, characterized by the initial immune response to EBV, was defined by specific serological profiles (VCA IgM+/VCA IgG−/EBNA IgG− or VCA IgM+/VCA IgG+/EBNA IgG−) and additional criteria, as outlined in Figure 2, to minimize false positives. In the presence of positive VCA IgG, positive VCA IgM, and negative EBNA IgG, primary infection was retained in the absence of exclusion criteria. On the other hand, non-specific VCA IgM is relatively common in our practice. Therefore, primary infection was only considered if the clinical inclusion criteria were met in cases where VCA IgM was the only positive marker. Active infection, which would require evidence of viral replication such as positive PCR results, was not specifically assessed in this study. In terms of other phases of infection, reactivation was not assessed in our analysis because of the lack of clear criteria to distinguish between a transient state following primary infection and reactivation of latent infection, especially given the retrospective nature of the study.

### 2.3. Statistical Analysis

All data were analyzed using GraphPad Prism 8.0.2 software (for Windows, GraphPad Software, Boston, MA, USA). For quantitative variables, we used *t*-tests for pair-wise comparisons or ANOVA for multiple comparisons, followed by Tukey’s multiple-comparison test when there was a significant difference. Categorical variables were compared using chi-square tests. The level of significance was set at 0.05. Other statistical analyses were performed using R (version 4.3.2) [24]. The chi-square test was used for global comparisons, followed by pairwise post-hoc comparisons of proportions using the prop.test function; *p*-values were adjusted for multiple comparisons using the Bonferroni correction. The following R packages were used: readxl [25], writexl [26], and ggplot2 [27] for data handling, output, and visualization.

### 2.4. Ethical Considerations

This retrospective study was conducted in accordance with the Declaration of Helsinki, and the protocol was approved by the local institutional review board of the Hospital University Centre of Amiens Picardie (reference: PI2024_843_0053, 17 April 2024).

Informed consent for participation is not required according to our local legislation.

## 3. Results

### 3.1. Patient Characteristics

From January 2013 to December 2023, 19,771 patients were enrolled in the study (Figure 1); 17,794 results were retained after deduplication. Inclusion flowcharts are shown in Figure 1 and Figure 2, and patient characteristics are shown in Table 1. Among patients with a primary infection profile, 464 were retained as primary infections, while 207 were excluded and classified as doubtful. There was a significant increase in the number of requests for EBV serology between 2013 and 2016 (more than 16-fold), which then remained relatively stable, with only a moderate increase each year. The male to female ratio was 1.06 (9167/8627), ranging from 0.9 to 1.17 depending on the year.

**Figure 3:**
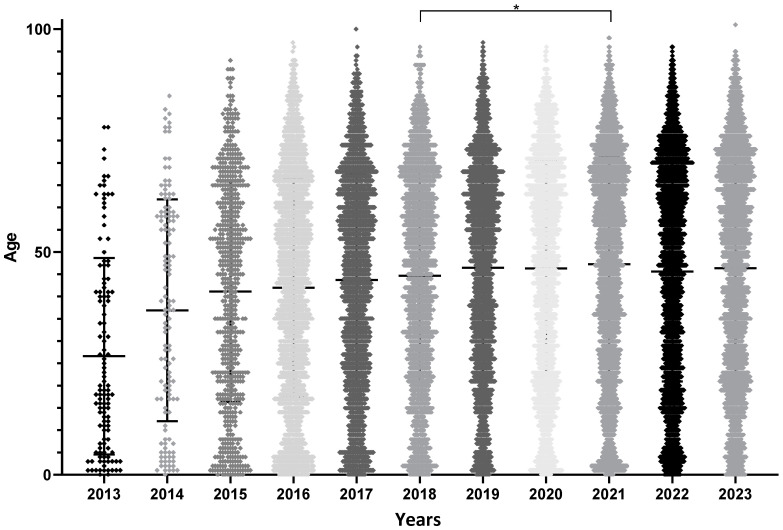
Age of screened patients by year of screening. ANOVA revealed significant differences (*p*-value < 0.0001); subsequent pairwise comparisons from 2013 to 2018 showed a progressive increase in the age of patients screened (significant differences with Tukey’s test). On the contrary, from 2018 onwards, no differences were found between any pair of years, except for 2018 vs. 2021 (* = *p*-value < 0.05).

There was no significant difference in the sex ratio during the 11 years (chi-square test, *p*-value = 0.08). The mean age ± standard deviation of the mean was 45.1 ± 0.2 years. As for the total number of screening tests, the mean age increased significantly until 2018 and then stabilized at approximately 45 years (Figure 3 and Table 1). ANOVA, followed by Tukey’s multiple-comparison test, showed significant differences before 2018 (*p*-values not displayed on the Figure 3 to avoid overloading it as most years differ pairwise before 2018) and non-significant differences in patient age between 2018 and 2023, except between 2018 and 2021, where patients tested in 2021 were significantly older. This could introduce a bias in further analyses. The units that prescribed EBV serology remained the same, with 25% of the tests corresponding to specialized medical consultations, the hematology unit, and follow-up of kidney transplant recipients. However, from 2016 onwards, these units accounted for a growing share of requests, to the detriment of those from pediatric wards, which partly explains the rapid ageing of patients included at the beginning of the study.

### 3.2. Global EBV Seroprevalence

**Figure 4:**
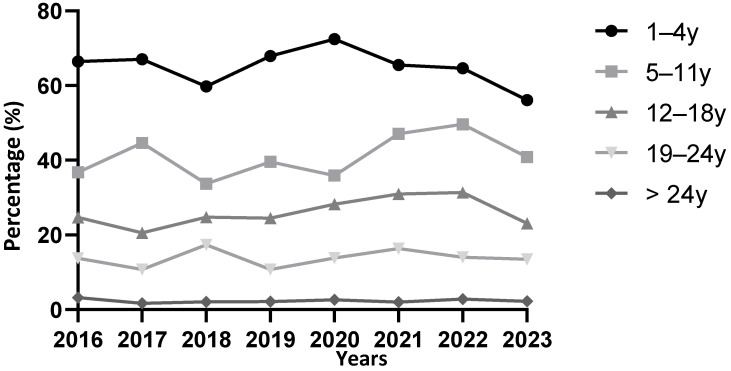
Proportion of EBV seronegativity by age group according to year of screening. Categories with fewer than 50 patients tested have been removed from the graph to avoid bias due to the small cohort size.

Among the 17,794 patients screened for EBV, the overall seronegativity was 10.2%, suggesting an overall seropositivity of approximately 90% if all patients with a positive serology are considered, regardless of their serological profile, with a past infection prevalence of 73.5%. The EBV seronegativity rate logically varied by age group, with no significant variation between the different years of the study (Figure 4). After 25 years of age, EBV seronegativity was consistently close to 0% and remained similar regardless of the year of testing.

The age of EBV seropositive patients increased significantly until 2018 due to an increase in the age of patients screened (ANOVA, *p*-value < 0.0001). There was no significant difference in the age of seropositive patients between 2018 and 2023, except between 2018 and 2021, where seropositive patients were older in 2021, although this may be related to the recruitment bias mentioned above.

### 3.3. Primary Infection

The overall mean seroprevalence of primary infections was 2.6%. Comparison of the proportions (using R 4.3.2) showed no significant differences between years since 2016, but there were significant differences between 2013, 2014, and possibly 2015 and the other years, consistent with the variation in screening indications (younger patients were screened more frequently from 2013 to 2015, so logically more tests for primary infection than in more recent years).

**Figure 5:**
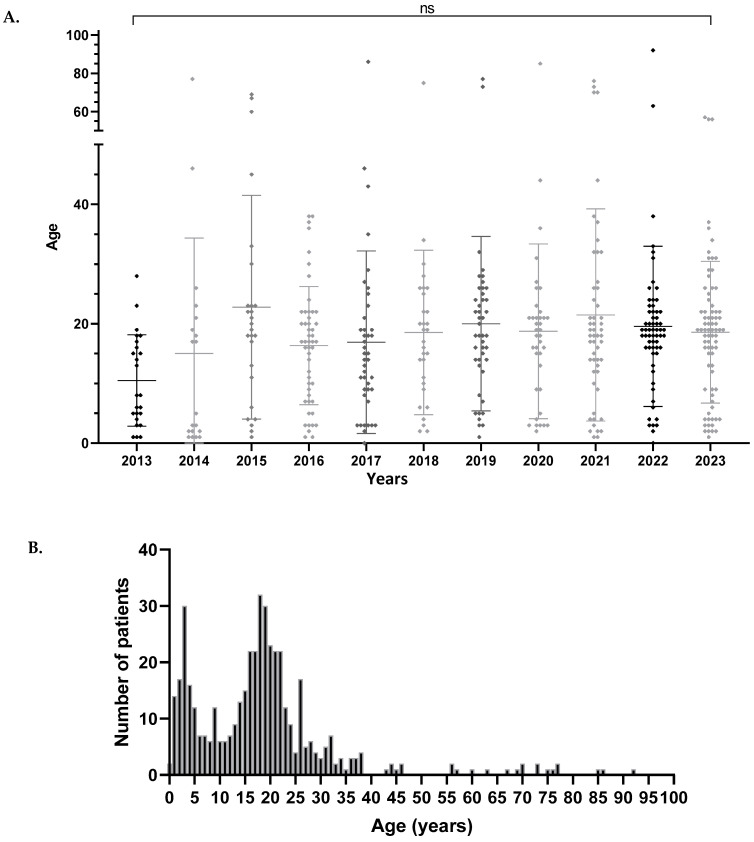
(**A**) Scatterplot of age of patients with primary EBV infection by year of serological testing. ANOVA shows no significant change in mean age over time (*p* > 0.05, ns = non-significant). (**B**) Mean age distribution of primary infections from 2013 to 2023.

There were no significant differences in the mean age of primary infection between years (Figure 5A) or sex (*t*-test, *p*-value = 0.2475). The evolution of the age of primary infection showed a bimodal distribution with peaks between the ages of one and five years, and between 15 and 23 years, with a dip during the primary and secondary school period (Figure 5B).

**Figure 6:**
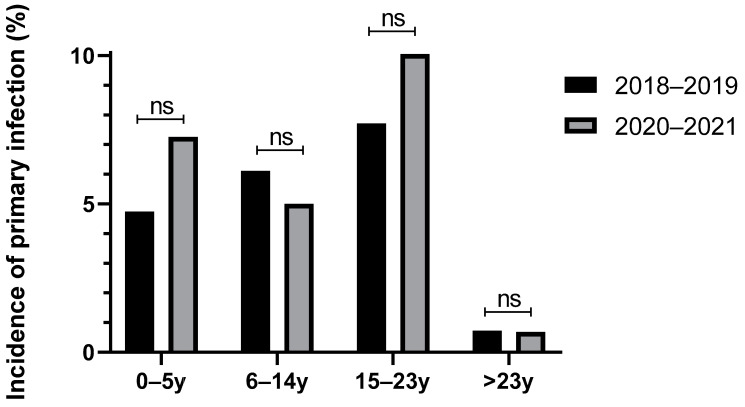
Incidence of primary infection by age group before and after COVID-19 onset, with pairwise comparison using Fisher’s exact test (ns = non-significant).

The data in Figure 6 specifically show the incidence of primary infections in the two years prior to the start of COVID-19 (2018–2019) compared to the two following years (2020–2021). This represents the percentage of children, among those tested, who had a profile consistent with a primary infection in these years. Notably, there was no significant difference in the age distribution of primary infections between these two periods. However, certain trends (although not statistically significant) were observed, with an increase in incidence among children aged 1–5 years (+2.5%) and adolescents aged 15–23 years (+2.3%).

## 4. Discussion

In this study, the seroprevalence of EBV and, in particular, the average age for the different serological profiles was examined from 2013 to 2023. The change in the age of patients screened until 2017 makes it difficult to interpret the average age for the different profiles due to recruitment bias. From 2018, the groups are more comparable as there are no significant differences between the main characteristics of the samples. These differences are explained by the evolution of the indications for EBV serological screening [21]. Indeed, the patients screened were older and far removed from the primary EBV infection, especially for screening prior to immunosuppressive therapy. This partly explains the predominance of hematology and nephrology units among the most frequent prescribers of EBV serological follow-up, which are more oriented toward pre-therapeutic serological status screening, whereas pediatric emergency or hepatogastroenterology units are more focused on identifying primary infections.

The overall seroprevalence of EBV in this cohort is comparable to the data available in the literature on the prevalence of EBV in France. For example, a study by Fourcade et al. conducted between 2000 and 2016 showed that the percentage of EBV-seronegative patients over 20 years of age varied over time from 2.1% to 3.1%, which is close to the seronegativity (<3%) observed here in patients over 25 years of age [8]. This national seroprevalence appears to be higher than the worldwide prevalence of approximately 90% in adults [4]. In contrast to the studies presented in the introduction, there appears to have been no decline in EBV seroprevalence in our cohort between 2013 and 2023, with overall stable seropositivity rates regardless of the age group.

The acquisition of primary EBV infection has been described as bimodal, with peaks before the age of six years and after the age of 10 years [28]. We observed a similar pattern, with peaks before the age of six years and between the ages of 15 and 23 years, which appears to correlate with different patterns of social interaction at these stages of life. Young children, particularly in larger families or in developing countries, tend to have frequent and close social contacts (within the family, day care, or pre-school) that favor the saliva-mediated transmission of EBV. In contrast, infectious mononucleosis is common when primary infection occurs later in adolescence, due to changes in social behavior such as kissing and sharing drinks, which increase the risk of transmission [12,13].

This observation draws attention to the role of social interactions in the epidemiology of EBV and raises the question of how the COVID-19 pandemic may have affected this dynamic. The COVID-19 pandemic had a major impact on public health, leading to numerous sanitary restrictions and measures to prevent respiratory transmission. Specifically, from 17 March 2020 to 30 June 2021, France experienced three lockdowns and several curfews. As a result of these strict hygiene measures, the transmission of many other pathogens, mainly other respiratory viruses, was reduced during the COVID-19 pandemic [29]. EBV is a *Herpesviridae* that is mainly transmitted via saliva [30], and it therefore seemed relevant to investigate the impact of the COVID-19 pandemic on its epidemiology. In our study, we did not observe a significant difference in the proportion of primary infections in 2020 compared with other years. Interestingly, there was a trend towards an increase in the incidence of primary EBV infections in the two most susceptible age groups: 1–5 years and 15–23 years. This suggests that the lockdown did not have a major impact on the age distribution of primary EBV infections. In young children, intra-familial transmission remains highly probable. However, it is surprising that the restriction of social contact among adolescents did not prevent primary infections, as we would expect their social circles to have been reduced during the pandemic. It is also important to consider that the confinement period disrupted the health care system, which may have led to an increase in targeted serological testing for infectious mononucleosis symptoms, while systematic screening may have been reduced as health services prioritized primary care. Nevertheless, the number of serologies performed was very similar between the two periods (see Table 1).

While our data show a trend towards increased primary infections among 15–23-year-olds between 2018–2019 and 2020–2021 (non-significant result), no significant decrease in EBV seropositivity was observed during this period. This contrasts with a study from China [11] that reported a 30% decrease in the absolute number of EBV-positive serologies between 2019 and 2020, suggesting a possible impact of the COVID-19 pandemic on EBV epidemiology. However, this study did not take into account the overall decrease in serology testing during the pandemic (12,582 tests in 2020 vs. 16,462 in 2019), which is likely to bias this result. In relative terms, the proportion of acute EBV infections among all serologies decreased only slightly (12.4% in 2019 vs. 11.5% in 2020). Interestingly, their data showed a significant decrease in EBV seropositivity among 1–3-year-olds in 2020–2021 compared to 2019. Although our study does not show a similar trend, these age-specific differences warrant further investigation. In addition, regional differences in healthcare interruptions and public health responses to COVID-19 suggest that findings from China may not be directly applicable to Europe. Our results showing no clear delay in primary EBV infection after the pandemic provide reassuring insights into EBV epidemiology, as delayed primary infection is associated with an increased risk of infectious mononucleosis, multiple sclerosis, and Hodgkin’s lymphoma. This highlights the importance of studying post-COVID EBV epidemiology in different geographical settings.

A major limitation of our study is that classical EBV serology primarily examines the immune response rather than directly reflecting viral replication or shedding, and therefore does not provide a direct indication of viral circulation. More precise approaches, such as serological testing for early antigen (EA) or immediate early antigen (IEA) antibodies, or PCR-based viral load measurements in saliva, could have provided further insight into the transmission and circulation dynamics of EBV. However, these methods were not available in the context of this retrospective study. In addition, although our cohort was large, it was monocentric and included only patients seen in consultation or hospitalized for a wide range of pathologies. In addition, the relatively small number of primary infections may have limited the statistical power, possibly preventing the detection of any effect that may exist. Although our results are reassuring regarding the limited effect of COVID-19 on EBV seroprevalence and associated risk of pathology, multicenter studies with more primary infections would be valuable to confirm our findings.

## 5. Conclusions

Over the past 10 years, EBV serological profiles have remained stable, with no significant changes in the overall dynamics of EBV epidemiology. The observed variations in results are mainly due to changes in the indications for screening, in particular, the increasing focus on pre-therapeutic testing, rather than shifts in EBV transmission patterns. Although the COVID-19 pandemic did not significantly disrupt overall EBV transmission, it appears to have led to a slight increase in the incidence of primary infection among young children and adolescents, despite the reduction in social interactions during the lockdowns. Given the potential association of late primary infections with diseases such as multiple sclerosis and Hodgkin’s lymphoma, monitoring the long-term effects of these trends in larger cohorts may be warranted.

## Figures and Tables

**Table 1:** Patient characteristics.

	2013	2014	2015	2016	2017	2018	2019	2020	2021	2022	2023	Total
*n*	119	130	640	1982	1882	1895	1968	1806	2318	2528	2526	17,794
M/F	57/62	68/62	334/306	1028/954	983/899	956/939	1059/909	957/849	1214/1104	1276/1252	1235/1291	9167/8627
Past infection (%)	38.7	36.2	59.1	73.6	75.7	74.0	76.0	76.9	74.0	72.0	75.3	73.5
EBV negative (%)	15.1	14.6	13.1	12.5	9.8	9.2	8.8	10.5	9.5	11.2	8.6	10.2
Primary Infection (%)	20.2	14.6	4.1	2.5	2.2	1.6	2.2	2.1	2.3	2.5	3.0	2.6
Others (%)	26.1	34.6	23.8	11.5	12.3	15.1	13.0	10.5	14.2	14.4	13.1	13.7
Mean age (±standard deviation of the mean)	26.6 ± 2	36.9 ± 2.2	41.1 ± 1	42 ± 0.5	43.7 ± 0.5	44.7 ± 0.5	46.5 ± 0.5	46.4 ± 0.6	47.3 ± 0.5	45.6 ± 0.5	46.4 ± 0.5	45.1 ± 0.2

## Data Availability

The original contributions presented in the study are included in the article, further inquiries can be directed to the corresponding author.
